# The effect of heterologous VHb expression to the functioning of stress-related genes in hybrid aspen lines exposured to biotic stress

**DOI:** 10.1186/1753-6561-5-S7-P88

**Published:** 2011-09-13

**Authors:** Hely Häggman, Suvi Sutela, Soile Jokipii-Lukkari, Tiina Ylioja, Risto Kasanen, Anna-Kaisa Anttila, Marko Suokas, Estelle Dumont, Pauli T Kallio

**Affiliations:** 1 Department of Biology, University of Oulu, Finland; 2 Finnish Forest Research Institute, Suonenjoki Research Unit, Finland; 3 Department of Forest Sciences, University of Helsinki, Finland; 4 Institute of Microbiology, ETH-Zürich, CH-8093 Zürich, Switzerland

## Background

The hemoglobin produced by *Vitreoscilla* bacterium, VHb, is capable to promote the respiratory activity and ATP production under hypoxic conditions [1]. In micro-organisms grown under oxygen-limited conditions, the heterologous *vhb* expression improves the production of primary and secondary compounds, growth as well as stress tolerance [2,3]. VHb has also been shown to protect plants against deleterious effects of nitric oxide (NO), the important signalling molecule produced during pathogen challenges [4]. We have previously studied the *vhb* expressing hybrid aspen lines (*Populus tremula* L. x *tremuloides* Michx.) under elevated UV-B illumination and showed that the VHb lines had increased secondary metabolite production and also enhanced accumulation of starch in chloroplasts when grown in standard greenhouse conditions [5]. In order to reveal the effect of VHb on the expression of stress-related genes in hybrid aspen exposed to biotic stress, we conducted experiments with pathogenic fungus and larvae of insect herbivore.

## Materials and methods

In the herbivory experiment, two-year-old VHb line and non-transgenic hybrid aspen line were exposed to the feeding of chestnut moths larvae (*Conistra vaccinii* L.) for 24 hours. Four VHb hybrid aspen lines and two non-transgenic hybrid aspen lines were infected with pathogenic fungus *Venturiatremulae*, *in vitro*. Samples for the RNA and protein extractions were collected after 10 and 21 days of the pathogen inoculation. In both experiments, the transcriptome changes were studied with microarrays and real-time RT-PCR. The cDNA slides contained 8256 stress-related oligos of *Populus euphratica.* The relative expression of selected genes were conducted with the LightCycler® 2.0 (Roche) and LightCycler® 480 instruments according to the manufacturer’s chemistry. The amount of VHb protein during pathogen fungus experiment was analysed by Western blots.

## Results and conclusions

The consumption of the leaves by the chestnut moth larvae was comparable on the non-transgenic and the VHb line. The effect of herbivory to the gene expression levels of wounded and systemic leaves was similar in the VHb and non-transgenic hybrid aspen lines, but in the non-transgenic line, the detected changes were more severe than in the VHb hybrid aspen line. The genes encoding bark storage proteins (BSPs), copper chaperone (CCH), and nitrate transporter (NRT) were among the limited number of genes which showed different expression changes between the VHb and non-transgenic hybrid aspen line.

The hybrid aspen lines infected with *V. tremulae* showed symptoms of shoot blight i.e. blackened stems and leaves, but the amount of VHb protein did not correlate with the severity of the disease. At the transcriptome level, the studied lines responded to the pathogen treatment individually in the first sampling (10 days) whereas the expression profiles of the lines resembled each other in the second sampling (21 days). The functional grouping of the genes with known mode of action resulted most representatives in protein metabolism, photosynthesis, stress as well as RNA metabolism groups.

In general, the transcriptome profiling of fungus- and herbivory-induced responses did not reveal any biological or molecular process altered in all of the VHb lines due to the biotic interactions. Moreover, based on the real-time RT-PCR results, the *vhb* expression did not show induction after the 24-hour herbivore treatment or the inoculation with the *V. tremulae*.
